# The Effect of Human Milk Oligosaccharides and *Bifidobacterium longum* subspecies *infantis* Bi-26 on Simulated Infant Gut Microbiome and Metabolites

**DOI:** 10.3390/microorganisms11061553

**Published:** 2023-06-10

**Authors:** Krista Salli, Johanna Hirvonen, Heli Anglenius, Ashley A. Hibberd, Ilmari Ahonen, Markku T. Saarinen, Johanna Maukonen, Arthur C. Ouwehand

**Affiliations:** 1 Global Health & Nutrition Science, IFF Health, 02460 Kantvik, Finland; johanna.hirvonen@iff.com (J.H.); heli.anglenius@iff.com (H.A.); markku.saarinen@iff.com (M.T.S.); pia-johanna.maukonen@iff.com (J.M.); arthur.ouwehand@iff.com (A.C.O.); 2 Genomics & Microbiome Science, IFF Health, Madison, WI 53716, USA; ashley.hibberd@iff.com; 3 Vincit Oy, 20500 Turku, Finland; ahosen.ilmari@gmail.com

**Keywords:** infant colon simulations, human milk oligosaccharides, probiotics, *Bifidobacterium longum* subspecies *infantis* Bi-26, 2′-fucosyllactose, 3-fucosyllactose

## Abstract

Human milk oligosaccharides (HMOs) shape the developing infant gut microbiota. In this study, a semi-continuous colon simulator was used to evaluate the effect of 2 HMOs—2′-fucosyllactose (2′-FL) and 3-fucosyllactose (3-FL)—on the composition of infant faecal microbiota and microbial metabolites. The simulations were performed with and without a probiotic *Bifidobacterium longum* subspecies *infantis* Bi-26 (Bi-26) and compared with a control that lacked an additional carbon source. The treatments with HMOs decreased α-diversity and increased *Bifidobacterium* species versus the control, but the *Bifidobacterium* species differed between simulations. The levels of acetic acid and the sum of all short-chain fatty acids (SCFAs) trended toward an increase with 2′-FL, as did lactic acid with 2′-FL and 3-FL, compared with control. A clear correlation was seen between the consumption of HMOs and the increase in SCFAs (−0.72) and SCFAs + lactic acid (−0.77), whereas the correlation between HMO consumption and higher total bifidobacterial numbers was moderate (−0.46). Bi-26 decreased propionic acid levels with 2′-FL. In conclusion, whereas infant faecal microbiota varied between infant donors, the addition of 2′-FL and 3-FL, alone or in combination, increased the relative abundance and numbers *Bifidobacterium* species in the semi-continuous colon simulation model, correlating with the production of microbial metabolites. These findings may suggest that HMOs and probiotics benefit the developing infant gut microbiota.

## 1. Introduction

The establishment of the microbiota in early life also shapes one’s health later in life [1,2,3]. Several factors, such as delivery mode, diet, medication, and environment, influence the composition and numbers of the developing microbiota in infants [2,4]. As an abundant genus in infancy, *Bifidobacterium* is critical to the development of the infant gut microbiota, in contrast to the adult microbiota, which primarily harbours other bacteria from the phyla Firmicutes and Bacteroidetes [5]. The ability of certain *Bifidobacterium* species to utilise oligosaccharides from human milk increases their colonisation and abundance [5,6]. *Bifidobacterium* species also support host-microbiota homeostasis [1].

In addition to live commensal bacteria, human breast milk contains bioactive compounds, such as human milk oligosaccharides (HMOs), a group of structurally diverse carbohydrates [2,7]. HMOs are not hydrolysed by intestinal enzymes in the upper gastrointestinal tract [8] and promote the growth of certain bacteria in the colon, such as *Bifidobacterium longum* subsp. *infantis* (*B. infantis*), *Bifidobacterium bifidum*, and several *Bacteroides* species, resulting in the production of microbial metabolites that typify carbohydrate fermentation [3,9,10,11].

The composition of breast milk is dynamic and adapts to the needs of the growing infant [12]. Breast milk is the recommended nutrition for infants, but for various reasons, breastfeeding is not always possible. Some HMOs that are abundant in breast milk are also available through large-scale commercial production, including 2′-fucosyllactose (2′-FL) and 3-fucosyllactose (3-FL). Supplementing a milk substitute with 2′-FL and/or *B. infantis* Bi-26 has been shown to be safe in a piglet study [13]. In addition, 2′-FL, *B. infantis* Bi-26, and their combination supported piglet growth with no disadvantageous effects on body and organ weight or intestinal structure and function [13]. Furthermore, 2′-FL has been clinically studied as a component of infant formulas in healthy, full-term infants—alone [14,15], in combination with *Limosilactobacillus reuteri* DSM 17938 [16] or lacto-N-neotetraose (LNnT) [17], and as part of 2 5-HMO blends: 2′-FL, 2′,3-di-fucosyllactose (DFL), lacto-N-Tetraose (LNT), 3′-sialyllactose (3′-SL) and 6′-sialyllactose (6′-SL)) [18] and 2′-FL, 3-FL, LNT, 3′-SL and 6′-SL [19,20]. In contrast, 3- FL has only been studied as part of a 5-HMO blend [19,20]. These studies have found HMOs to be safe and well tolerated and to support the growth of infants by age. In clinical trials, supplementation with HMOs has modulated the composition of the infant gut microbiota to more closely approximate that of the breast-fed reference group—for example, by increasing *Bifidobacterium* levels [16,18,21].

In vitro data have shown that 2′-FL and 3-FL are utilised in pure-culture by *B. infantis* strains, including *B. infantis* Bi-26 [10,22,23,24]. In addition, *B. infantis* Bi-26 has been suggested to utilise fucosyllactose faster than the type strain *B. infantis* ATCC 15697 (DSM 20088) [24]. The effects of 2′-FL on the composition of complex microbiota have been studied in infant microbiota using batch cultivation, SHIME (ProDigest, Ghent, Belgium), and EnteroMIX^®^ (International Flavors & Fragrances, Kantvik, Finland) colon simulators [25,26,27,28,29]. Batch cultivation and the CoMiniGut model (University of Copenhagen, Copenhagen, Denmark) have been used to evaluate the effects of 3-FL on the infant microbiota [30,31]. The composition of the infant microbiota varies widely between infants who donate the faecal inoculum, introducing variability in infant simulations and affecting the efficiency of HMO utilisation [25,26,27,28,31,32].

According to a recent review, supplementation with probiotics (or prebiotics and synbiotics) may beneficially affect the composition of the gut microbiota in infants who are born by caesarean section [33]. *Bifidobacterium* levels approached those of vaginally born infants, especially those who were breast-fed [33]. However, the results differed between probiotic strains and doses, alone and in combination with prebiotics, necessitating greater insight into the effects of individual HMOs with and without probiotics.

In this study, bacterial fermentation of the infant colon was modelled using the EnteroMIX^®^ colon simulator [26,34,35,36,37]. Our aim was to compare 2′-FL and 3-FL—alone, in combination, and with the probiotic *B. infantis* Bi-26—with regard to their effects on the diversity, composition, and metabolic activity of the infant gut microbiota in vitro. Although the compositions of the simulated infant faecal microbiota varied between infant donors, the addition of 2′-FL and 3-FL alone and in combination increased the relative abundance and numbers of *Bifidobacterium* species and was accompanied by the production of microbial metabolites.

## 2. Materials and Methods

### 2.1. EnteroMIX^®^ Colon Simulator Model

For modelling the infant gut microbiota, frozen infant faecal samples were used to prepare an inoculum for the colon simulator system. Three donor infants, all aged under 4 months, were in good health and had not been medicated with antibiotics. A parent of each infant gave informed consent and provided background information on the infant who donated the faecal sample with regard to age, diet, supplements, allergies, and mode of delivery. The instructions and equipment for sample collection were provided to the parents. The faecal samples were frozen immediately at home using Oxoid™ AnaeroGen™ (Basingstoke, UK) products (CO_2_Gen Compact, plastic pouches, and compact sealing clips) to limit their exposure to oxygen before being collected. This study was reviewed and approved by the Coordinating Ethical Committee of the University of Helsinki (Decision number 139/13/03/00/16). All methods were performed in accordance with the national guidelines of Finland.

**Figure 1:**
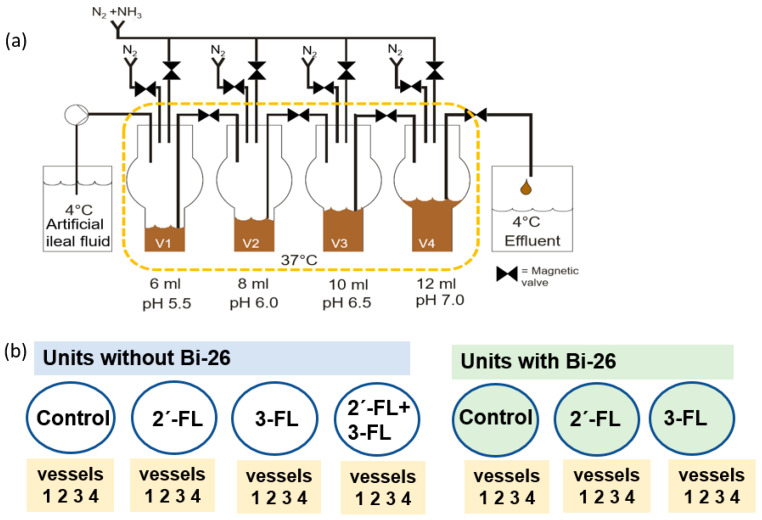
Schematic of the EnteroMIX^®^ colon simulator and overview of the study products. (**a**) A diagram of a single unit of the EnteroMIX^®^ colon simulator system. Vessel 1 (V1, proximal) to V4 (distal) represent various parts of the colon. Nitrogen was used to maintain anaerobiosis and as a carrier gas for ammonia and liquid transfers. The system was computer-controlled (figure modified from [37]). (**b**) An overview of the treatments in a single simulation. Three simulations were performed. Control = no FLs; 2′-FL = 2′-fucosyllactose; 3-FL = 3-fucosyllactose; Bi-26 = *Bifidobacterium longum* subspecies *infantis* Bi-26.

The 4-stage semi-continuous EnteroMIX^®^ colon simulator model was used to study the effects of 2′-FL, 3-FL, 2′-FL + 3-FL, 2′-FL + *B. infantis* Bi-26, 3-FL + *B. infantis* Bi-26, and *B. infantis* Bi-26 alone on the infant intestinal microbiota [35,36,37,38]. Artificial ileal fluid was used as the medium in this simulator [38] as it best represents the liquid coming from the small intestine into the colon. 2′-FL (CARE4U^®^, International Flavors & Fragrances, New York, NY, USA) and 3-FL (Inbiose NV, Ghent, Belgium and IFF, Kantvik, Finland) were suspended in artificial ileal fluid to serve as the carbon source, whereas artificial ileal fluid alone was fed to the system for the control simulations. Three simulations, using faecal samples from 3 different infants as inocula, were performed as previously reported [26]. Each simulation had 7 parallel treatments (Figure 1); 2′-FL and 3-FL were studied at 2% (*w*/*v*), as was the combination of 2′-FL + 3-FL (so 2′-FL and 3-FL each constituting 1% (*w*/*v*), and the overall HMO content was 2% also with 2′-FL + 3-FL).

Before the simulation began, 10 mL of individual inoculum was pumped into the first vessel, mixed, transferred to the second vessel, mixed, transferred to the third vessel, mixed, transferred to the fourth vessel, and mixed; the effluent was discarded. In the units with *B. infantis* Bi-26 (ATCC SD6720), 1 mL of an overnight culture of *B. infantis* Bi-26 (10^9^ cells/mL) was added to 9 mL of inoculum (~1 × 10 ^10^ cells/mL); *B. infantis* Bi-26 thus constituted approximately 1% of the microbes inoculated in the simulator. Samples from the inocula with and without *B. infantis* Bi-26 were drawn to determine their microbial composition.

The test products (2′-FL, 3-FL, 2′-FL + 3-FL) or controls (without added carbohydrates) were fed to the simulator system at 3 h intervals during the simulation, over a total of 48 h. Then, the samples were collected from the vessels, and the composition of the simulated microbiota and microbial metabolites was analysed.

### 2.2. Quantification of Fucose, 2′-FL and 3-FL

Fucose, 2′-FL, and 3-FL were quantified from the simulation units to which HMOs were added as per Salli et al. [26] with modifications. Standard solutions of fucose (Sigma-Aldrich, St. Louis, MO, USA), 2′-FL, and 3-FL were prepared in water to concentrations of 80, 60, 40, 20, and 10 mg/l and stored at +4 °C. Sample solutions were centrifuged at 16,000× *g* for 5 min; then, 50 μL of the supernatant and 200 μL of ethanol were mixed in a microcentrifuge tube and incubated at +4 °C for 30 min. After centrifugation at 16,000× *g* for 5 min, 200 μL of the supernatant was evaporated to dryness at 30 °C under stream of nitrogen, and the solid residue was dissolved in 2000 μL of water and filtered. Separation and detection of the analytes were performed using high-performance anion-exchange chromatography as previously described [26]. Retention times of fucose, 2′-FL, and 3-FL were 6.3 min, 26 min, and 18 min, respectively.

### 2.3. Total Bacterial Cell Counts

Total bacterial cell counts were determined by flow cytometry in samples that were fixed with 4% formaldehyde, as described [39].

### 2.4. Quantitative Polymerase Chain Reaction (qPCR)

Microbial DNA was extracted from the colon simulation samples, and total bifidobacterial numbers were analysed by real-time quantitative polymerase chain reaction (qPCR) as previously reported [26,36,40]. *B. infantis* was quantified by qPCR using TaqMan and Applied Biosystems Real-Time PCR equipment and software (ABI 7500 FAST, Applied Biosystems, Foster City, CA, USA) with the Bi26_F (400 nM GTCACGATGTCTCCTTTGATATCAGCATG) and Bi26_R primers (400 nM CCTTTTGCGTCTCCCCCG) and the Bi26_P probe (200 nM, TCATTCATTGTAGTGGCGATCACCGTTACC). The annealing step for *B. infantis* was 60 °C for 30 s. Standard curves, consisting of 10-fold dilutions of target species DNA, were used for the quantification.

### 2.5. Microbial Composition by Barcoded 16S rRNA Amplicon Sequencing

The V4 variable region of the 16S rRNA gene was PCR-amplified from donor inoculum samples and control and the treated samples after 48 h of simulation, as previously described [41]. The amplicon pool was sequenced on the Illumina MiSeq system with 2 × 250 bp reads (Roy J. Carver Biotechnology Center, University of Illinois Urbana-Champaign, Champaign, IL, USA) and analysed using the Quantitative Insights Into Microbial Ecology pipeline (QIIME2, v.2018.6) [41,42]. In brief, the sequences were demultiplexed, and DADA2 was used to denoise and dereplicate the sequences. Representative amplicon sequence variants (ASVs) were assigned taxonomy against the Greengenes database (v. 13.8) [43]. Taxa compositions were reported as relative abundance (% of total sequences).

### 2.6. Analysis of Microbial Metabolites

The concentrations of short-chain fatty acids (SCFAs), lactic acid, and branched-chain fatty acids (BCFAs) in infant colon simulation samples were analysed by gas chromatography, as described by Ouwehand et al. [44].

### 2.7. Statistical Analysis

Alpha diversity comparisons were calculated in QIIME2 for the Phylogenetic Diversity (PD) Whole Tree metric [45] using an ASV table, rarefied at a sequence depth of 11,672. The main effect of treatment was analysed by Kruskal–Wallis test and corrected by the Benjamini–Hochberg false discovery rate (FDR); subsequent pairwise comparisons were conducted by Wilcoxon rank-sum test [46]. Beta diversity was calculated using weighted UniFrac values [47] and visualised by principal coordinates analysis (PCoA) in QIIME2. Differentially abundant taxa (>0.1% abundance) were determined by Kruskal–Wallis test, and *p*-values ≤ 0.05 were reported after adjustment using the FDR.

The remaining analyses were performed in R [48].

To determine the effects of *B. infantis* Bi-26 and HMOs on total bifidobacterial numbers by qPCR, a linear model with Gaussian error was used. First, the response was log2-transformed to better fit the modelling requirements, and a second-order term was included to account for nonlinear curves. Through an automated model selection process, the parameters were narrowed down to vessel, vessel squared, treatment group, *B. infantis* Bi-26, and the slope that was associated with *B. infantis* Bi-26, all of which were included in the model. The effect of *B. infantis* Bi-26 and the treatments on *B. infantis* were also analysed in a somewhat similar manner using log2-transformed response and a fixed effect model with terms for donor, treatment, and interaction between donor and vessel (i.e., donor-wise slope).

Differences in metabolite concentration curves across all vessels were analysed using nonparametric and robust methods by Brunner et al. [49] implemented in the R package nparLD [50]. This method allows us to analyse the variably shaped curves in a consistent fashion with minimal distribution assumptions. The effects of the treatment versus control on metabolites in the pooled vessels were analysed by student’s *t*-test. All *p*-values were corrected for FDR using the Benjamini–Hochberg method [46]. *p*-values of 0.05 or less were considered statistically significant.

The dependence between changes in 2′-FL, 3-FL and fucose, total bifidobacterial, and SCFA and BCFA counts was analysed by non-parametric Spearman correlation. The correlation estimates and their 95% confidence intervals were obtained using bias-corrected and accelerated bootstrap using 5000 samples. For illustration purposes, local polynomial regression fitting was used.

## 3. Results

### 3.1. Donor Demographics

Of the three faecal sample donors, Donors I and II were exclusively breast-fed, and Donor III was given breast milk and formula. None of the donors had begun consuming solid foods. Donor II was delivered by caesarean section, and donors I and II were delivered vaginally; all three were given vitamin D supplements, and Donors I and II were administered probiotics. When donating the faecal samples, Donor I was 3 months, Donor II was 3,5–4 months, and Donor III was 1 month old.

### 3.2. Fermentation of HMOs during Colon Simulation

The utilisation of 2′-FL and 3-FL by complex bacterial communities varied between simulations (Table 1). Table 1 also reports the fucose levels.

The addition of *B. infantis* Bi-26 resulted in more efficient 2′-FL utilisation in the simulations with Donor III. In simulations with Donors I and II, 2′-FL was utilised in the upstream vessels without *B. infantis* Bi-26. Conversely, the addition of *B. infantis* Bi-26 resulted in earlier and later 3-FL utilisation in the simulations with Donors II and I, respectively; this utilisation did not change considerably with Donor III. When 2′-FL and 3-FL were combined, their utilisation by the microbiota was similar in the respective simulations; with this combination, 2′-FL was used in downstream vessels only in simulation with Donor II. Overall, all three simulations consumed all 2′-FL prior to vessel 3; in the simulation with Donor III, this pattern was observed only with *B. infantis* Bi-26. In general, 3-FL was utilised before vessel 4, which occurred in simulation II only with *B. infantis* Bi-26.

After complete utilisation of HMOs, the amount of fucose, a downstream metabolite, was undetectable. However, when combining all simulations, the addition of *B. infantis* Bi-26 had a statistically significant difference on fucose levels in treatments with 2′-FL (*p* = 0.001), based on nonparametric analysis. The difference in simulations with 2′-FL was due to the addition of *B. infantis* Bi-26, which was different in three simulations (Table 1).

### 3.3. Microbiota Composition

#### 3.3.1. Microbial Composition of Inoculum Originating from Faecal Sample Used in the In Vitro Colon Simulator

**Figure 2:**
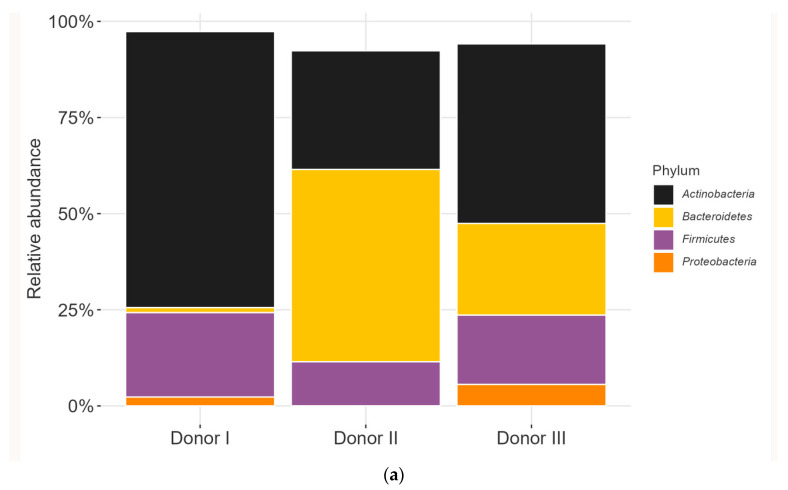

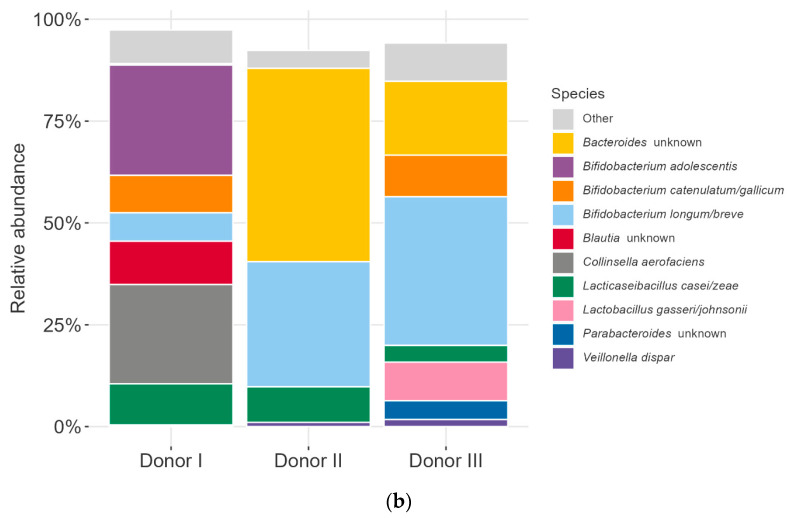
Bacterial composition of the inocula. The inoculum from each donor at the (**a**) phylum and (**b**) genus/species levels, with the 10 most abundant species shown.

By barcoded 16S rRNA amplicon sequencing, we noted clear differences in microbial populations between the three donor inocula (Figure 2 and Supplementary Table S1). Actinobacteria and Bacteroidetes were the most abundant phyla in all three donors (Figure 2a), totalling 70% in relative abundance; Firmicutes constituted between 10% and 20%, versus Proteobacteria at under 6% relative abundance.

At the genus/species level (Figure 2b), only the inoculum from Donor I contained *Bifidobacterium adolescentis*, *Collinsella aerofaciens*, and the genus *Blautia*, at relative abundances of ~27%, 24%, and 10%, respectively. The microbiota composition in the inoculum from Donor II differed from the other two donors, based on the presence of *Veillonella parvula* and the genus *Enterococcus* at relative abundance of ~0.8% for both. It also had the highest relative abundance of the genus *Bacteroides* (47%; species unidentified but determined not to be *Bacteroides fragilis*, *Bacteroides ovatus*, or *Bacteroides uniformis*).

The inoculum from Donor III differed from that of the other two donors, harbouring *Lactobacillus gasserii/johnsonii*, *Ligilactobacillus salivarius*, and *Limosilactobacillus vaginalis* at relative abundances of ~8%, 0.01%, and 2%, respectively. The inocula from Donors I and III contained *Bifidobacterium catenulatum/gallicum*, the family Enterobacteriaceae, and the genera *Parabacteroides* and *Phascolarctobacterium* (relative abundances: Donor I 9%, Donor III 10%; Donor I 2%, Donor III 6%; Donor I 0.1%, Donor III 5%; Donor I 0.1%, Donor III 0.5%, respectively), which were absent in Donor II.

#### 3.3.2. Alpha and Beta Diversity of Simulated Microbiota

**Figure 3:**
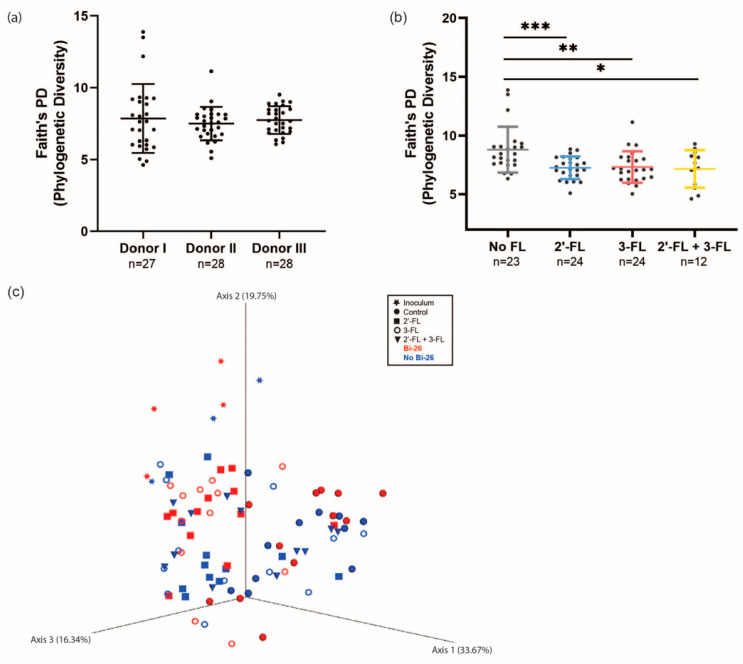
Alpha and beta diversity of the simulation samples. Alpha diversity (Faith’s phylogenetic diversity metric, with lines showing mean +/− SD) between (**a**) the three simulations (donors) and (**b**) treatment groups. (**c**) Beta diversity (weighted UniFrac metric), reflecting clustering of the microbiota samples by treatment, with or without *Bifidobacterium longum* subsp. *infantis* Bi-26 (Bi-26). No FL = control; 2′-FL = 2′-fucosyllactose; 3-FL = 3-fucosyllactose. Pairwise comparisons were conducted by Wilcoxon rank-sum test. *** *p* < 0.001, ** *p* < 0.01, * *p* < 0.05.

There was no difference in alpha (phylogenetic) diversity between donors (*p* > 0.1) (Figure 3a). Combining all three simulations, alpha diversity was greater in the control simulation compared with the treatments with HMOs (*p* < 0.05), and there was no difference between HMO treatment groups (*p* > 0.1) (Figure 3b). When comparing all study groups, the control group with *B. infantis* Bi-26 was significantly more diverse than the other groups (*p* < 0.05, Supplementary Figure S1); however, alpha diversity did not differ between samples with and without *B. infantis* Bi-26 (*p* > 0.1, Supplementary Figure S2).

Figure 3c shows the beta diversity (weighted Unifrac metric) of the samples by treatment, with or without *B. infantis* Bi-26, by principal coordinates analysis (PCoA).

#### 3.3.3. Effects of HMOs and *B. infantis* Bi-26 on Simulated Microbial Composition

With regard to gut microbiota composition by barcoded 16S rRNA amplicon sequencing, the following phyla predominated when all three simulated infant microbiota samples were combined after a 48 h simulation: on average, Firmicutes (38% relative abundance), Actinobacteria (3%), Proteobacteria (24%), and Bacteroidetes (8%).

The effect of HMOs was evaluated, pooling all three simulations. All HMO treatments, when the vessels were combined, increased the Actinobacteria abundance compared with the control (*p* = 0.004), whereas Bacteroidetes and Proteobacteria relative abundance were higher in the latter (*p* = 0.006 and *p* = 0.031, respectively). At the species level, *B. longum/breve* was the only species that increased with HMOs (*p* = 0.053); conversely, *Leucobacter* spp., *Enterobacteriaceae* spp., *Rummeliibacillus* spp., and *Enterococcus* spp. were higher in the control (*p* = 0.034 for *Leucobacter* spp., *p* = 0.048 *Enterobacteriaceae* spp. and *p* = 0.053 for others).

**Figure 4:**
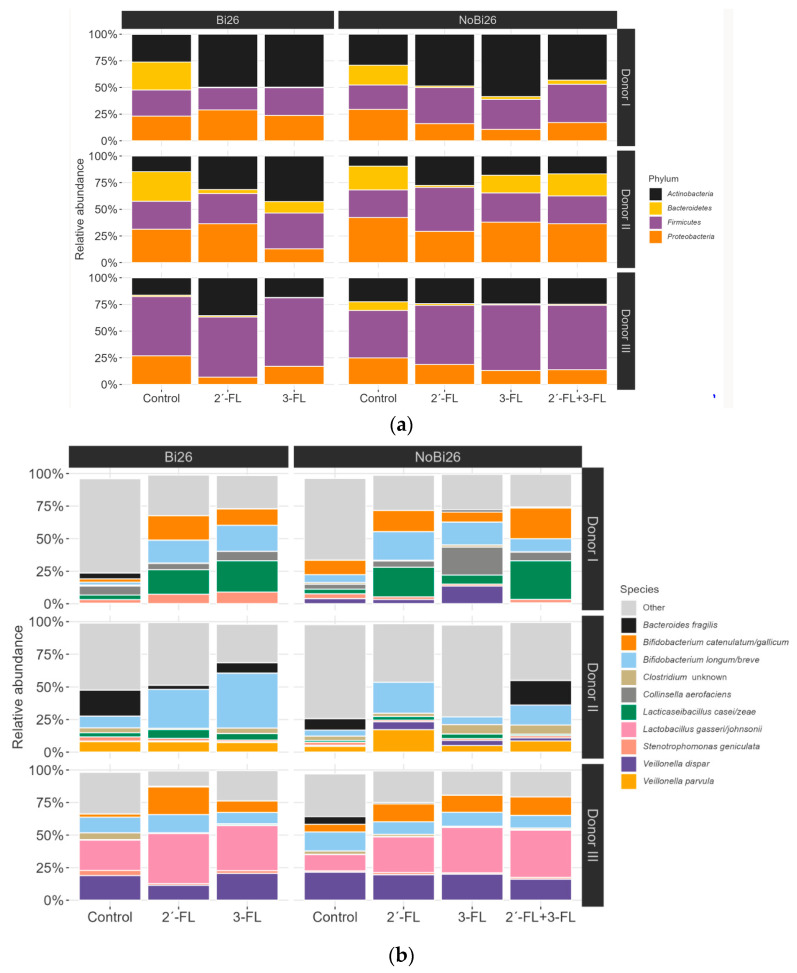

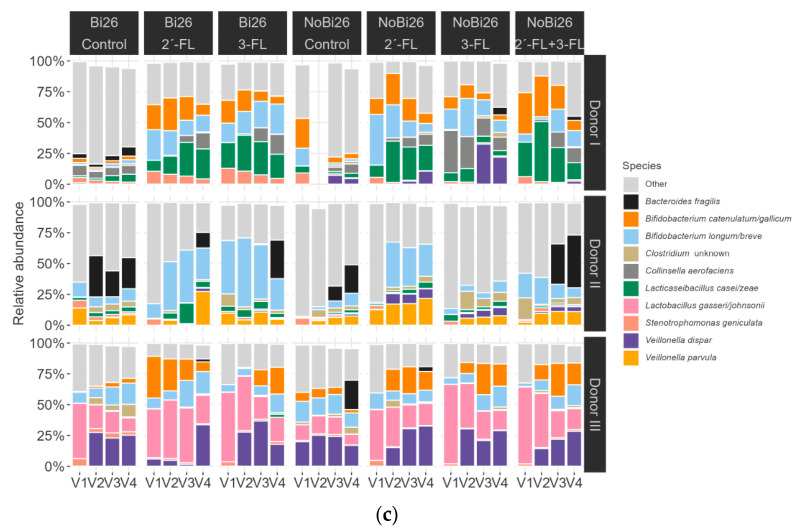
Microbial composition by barcoded 16S rRNA amplicon sequencing from simulation samples with and without *Bifidobacterium longum* subspecies *infantis* Bi-26 (Bi-26). Results at the (**a**) phylum level with all vessels pooled; (**b**) species level with all vessels are pooled, with the 10 most abundant genera shown; and (**c**) the species level for each individual vessel, with the 10 most abundant genera shown. The control simulation did not contain added carbohydrates, whereas the treatments comprised 2′-fucosyllactose (2′-FL), 3-fucosyllactose (3-FL), or their combination. Note: The results for the Donor I V2 control treatment are missing, due to low-quality DNA, and were not included in the analysis.

Because the microbiota composition varied between simulations with different donors, we have presented the results separately for each simulation. Figure 4 shows the microbial composition from the individual simulations and various treatments at the phylum (Figure 4a) and species levels (Figure 4b), with the vessels combined, and individual vessels at the species level (Figure 4c). At the species level, the most prominent change in the simulation with Donor I was the increase in relative abundance of *B. longum/breve*, *B. catenulatum/gallicum*, and *Lacticaseibacillus casei/zeae* on treatment with HMOs versus the control. In addition, in this simulation, the relative abundance of *Veillonella dispar* was higher when *B. infantis* Bi-26 was not added. In the simulation with Donor II with *B. infantis* Bi-26, the relative abundance of *B. longum/breve* rose with 2′-FL and 3-FL, and *Bacteroides fragilis* decreased compared with control. In the simulation with Donor II without *B. infantis* Bi-26 the relative abundance of *B. longum/breve* increased with 2′-FL and 2′-FL + 3-FL, while with 3-FL it did not. The relative abundance of *B. fragilis* increased in downstream vessels with 2′-FL + 3-FL. In the simulation with Donor III, relative abundance of *B. catenulatum/gallicum* increased between HMO treatments and control. In addition, there was increased relative abundance of *L. gasserii/johnsonii* with all HMO treatments. An Excel spreadsheet with all 16S rRNA amplicon sequencing results can be found in Supplementary Table S2: 16S rRNA amplicon sequencing.

#### 3.3.4. Total Bacterial, Total Bifidobacterial, and *B. infantis* Numbers of Simulated Microbiota

Based on the flow cytometry results, the total bacterial cell numbers increased from Vessels 1 to 4 in all units, in the control and HMO treatments, with and without *B. infantis* Bi-26 (Supplementary Table S3 Total bacterial cell numbers).

**Figure 5:**
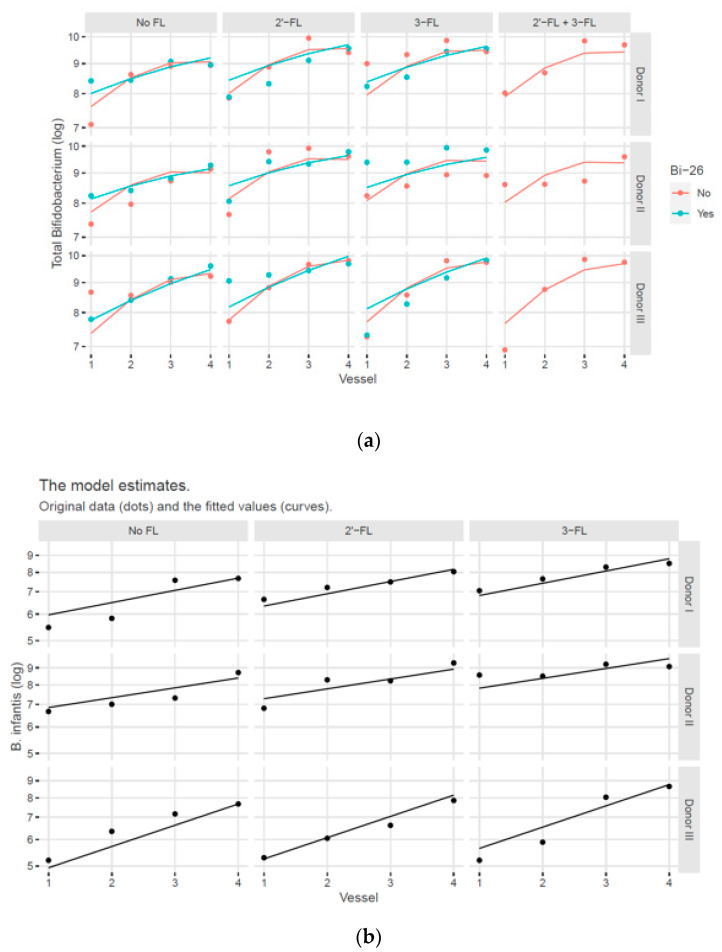
Total *Bifidobacterium* and *B. infantis* numbers by qPCR. (**a**) Total *Bifidobacterium* numbers from simulation samples with and without *Bifidobacterium longum* subspecies *infantis* Bi-26 (Bi-26) using the estimated curves for the individual donors and treatment groups. (**b**) *Bifidobacterium longum* subspecies *infantis* numbers by qPCR from the simulation vessels to which Bi-26 was added, using the estimated curves for the individual donors and treatment groups. Control simulation did not contain HMOs, whereas the treatment comprised 2′-fucosyllactose (2′-FL), 3-fucosyllactose (3-FL), or their combination. Pairwise comparisons between FL treatment groups were obtained by computing contrasts using the fitted statistical model. The original data *Bifidobacterium* numbers are shown as dots, and the fitted values are shown as curves. Control = no FL.

Total bifidobacterial and *B. infantis* numbers were analysed by real-time qPCR. Figure 5a shows the total *Bifidobacterium* numbers from simulation samples with HMOs, with and without *B. infantis* Bi-26. Vessel, vessel squared, treatment group, *B. infantis* Bi-26, and the slope that was associated with *B. infantis* Bi-26 were the main factors that contributed to the *Bifidobacterium* numbers and were included in the statistical model. In comparing the control against individual treatments, 2′-FL and 3-FL increased the overall bifidobacterial numbers by 5.3% and 4.6%, respectively.

Figure 5b shows a similar analysis of the *B. infantis* qPCR results in which only the simulations to which *B. infantis* Bi-26 was added were included. In simulations in which *B. infantis* Bi-26 was excluded, *B. infantis* numbers were below the limit of detection. Donor, treatment, vessel, and the interaction of donor and vessel were the main factors that contributed to the *B. infantis* numbers. Furthermore, 2′-FL and 3-FL increased *B. infantis* numbers versus control by 6.3% and 14.4%, respectively.

### 3.4. Microbial Metabolites

SCFAs (acetic acid, butyric acid, propionic acid), lactic acid, and BCFAs (2-methylbutyric acid, isobutyric acid and isovaleric acid) were measured to examine the effects of HMOs and *B. infantis* Bi-26 on bacterial metabolism. The effect for each metabolite was first analysed by subtracting the control values from the corresponding treatment values and pooling the vessels, such that the samples with and without *B. infantis* Bi-26 were considered separate. The 2′-FL + 3-FL combination was excluded because no *B. infantis* Bi-26 samples for this treatment were available. Next, the data were analysed to determine the overall effect of *B. infantis* Bi-26, examining the vessel level data using nonparametric methods per Brunner et al. [49].

#### 3.4.1. Effects of HMOs and *B. infantis* Bi-26 on Microbial Metabolite Production

**Figure 6:**
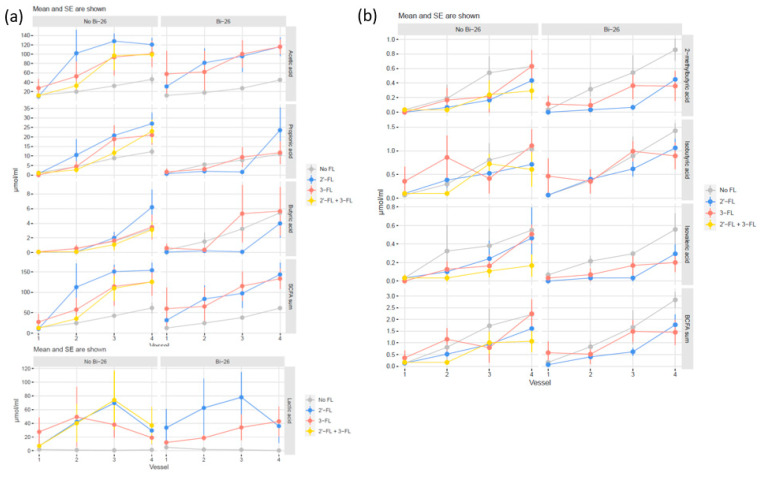
Short-chain fatty acid (SCFA) and branched-chain fatty acid (BCFA) production. (**a**) The production of SCFAs (acetic acid, butyric acid, propionic acid) and SCFAs and lactic acid and (**b**) production of BCFAs (2-methyl butyric acid, isobutyric acid, and isovaleric acid) and total BCFAs combined for three simulations but shown separately for 2′-FL and 3-FL treatments with and without *Bifidobacterium longum* subspecies *infantis* Bi-26 (Bi-26). No FL = control; 2′-FL = 2′-fucosyllactose; 3-FL = 3-fucosyllactose.

The results showed that 2′-FL effected the biggest changes in general metabolite levels versus the control, but as there were only small number of simulations, these changes did not reach statistical significance (*p*-values above 0.05). However, we observed a trend for an increase in acetic acid (*p* = 0.064) and SCFA sum (*p* = 0.064) with 2′-FL and in lactic acid with both 2′-FL and 3-FL (*p* = 0.116 and *p* = 0.116, respectively) (Figure 6a).

The addition of *B. infantis* Bi-26 had a statistically significant impact on propionic acid in the 2′-FL treatment (*p* = 0.013) (Figure 6a). The changes in the other metabolites were not statistically significant for the other treatments.

In general, minor amounts of BCFAs were produced in the simulations, peaking at 1.14 µmol/mL, 1.74 µmol/mL, and 1.1 µmol/mL for 2-methylbutyric acid, isobutyric acid, and isovaleric acid, respectively; overall, BCFA levels were lower with the HMO treatments compared with the control (Figure 6b). Furthermore, 2′-FL decreased isovaleric acid (*p* = 0.064) and 2-methylbutyric acid (*p* = 0.064), albeit nearly statistically significant. The addition of *B. infantis* Bi-26 did not affect BCFA levels (Figure 6b).

#### 3.4.2. 2′-FL and 3-FL Utilisation Correlates with SCFA and Total Bifidobacterial Counts

**Figure 7:**
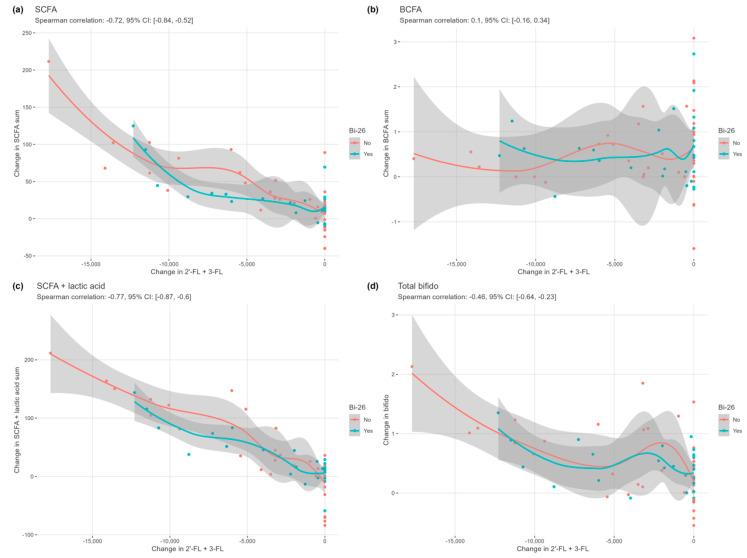
Correlation between human milk oligosaccharides, bacterial metabolites, and total bifidobacterial numbers. The correlation between changes in 2′-fucosyllactose (2′-FL) and 3-fucosyllactose (3-FL) and (**a**) short-chain fatty acids (SCFAs), (**b**) branched-chain fatty acid (BCFAs), (**c**) SCFAs + lactate, and (**d**) total bifidobacterial numbers.

We then examined how strongly the change in 2′-FL and 3-FL related to the changes in fucose, total bifidobacterial, SCFA, and BCFA levels, recorded from each transition between each consecutive vessel and each simulation. A clear relationship was seen between the utilisation in HMOs and the rise in SCFAs (−0.72, 95% CI: [−0.84, −0.52]) (Figure 7a) and SCFA + lactate (−0.77, 95% CI: [−0.87, −0.6]) (Figure 7c). The correlation between changes in HMOs and the increase in total bifidobacterial counts was moderate (−0.46, 95% CI: [−0.64, −0.23]) (Figure 7d). No association was seen for fucose (−0.26, 95% CI: [−0.52, 0.06]) (Supplementary Figure S3) or BCFAs (0.1, 95% CI: [−0.16, 0.34]) (Figure 7b).

## 4. Discussion

The early development of the infant gut microbiota is crucial for host–microbe interactions and in immediate and later human health [5,6]. *Bifidobacterium* is the dominant genus in the microbiota of healthy infants [5]. The ability of certain species of *Bifidobacterium* to utilise various HMO structures gives them a competitive advantage; conversely, the HMO composition can influence the microbiota composition [51]. Thus, it is important to better understand the interplay between bifidobacteria and HMOs. In this study, three in vitro colon simulations were performed with inocula from faecal samples from infant donors aged 1 to 4 months to determine the effects of 2′-FL, 3-FL, and their combination on the composition of the simulated infant microbiota and on the production of microbial metabolites. In addition, the probiotic *B. infantis* Bi-26 was examined in the presence and absence of 2′-FL and 3-FL.

The use of in vitro models is a valuable proxy for in vivo systems, particularly in infants, whose physiology and microbiota composition continue to develop with age. Moreover, there are still few in vivo data [52]. Thus, in vitro fermentation has gained recent interest regarding the effects of HMOs, probiotics, and their combinations [26,27,28,29,30,31,53,54,55]. Whereas certain studies have focussed on adult microbiota [31,53,54,55], others have analysed those in infants [25,26,27,28,30,31,32,56] and toddlers [28]. Several fermentation studies have used batch systems [25,28,30,32,53,55,56], and others have implemented more complicated systems [26,27,28,31,54,55], such as the in vitro simulated colon fermentation model in our study. The nutritional background media and other study parameters vary between the studies making direct comparisons challenging.

Because HMOs resist digestion and generally reach the colon intact [57], we used the EnteroMIX^®^ colon simulation model, which simulates human colonic fermentation, proceeding from the proximal to distal colon [26,34,37]. Our earlier study described its use in modelling infant colonic fermentation, finding that the simulations that were performed could be grouped by efficiency of 2′-FL utilisation indicating differences in the microbiota composition and HMO utilization capabilities [26]. Compared with our earlier work, which examined only 2′-FL, all three simulations in the current study were considered rapidly fermenting HMOs (2′-FL, 3-FL, or both) [26]. This was determined by complete or almost complete utilization of 2′-FL, 3-FL, or both within vessels 1 and 2 of the simulator, mimicking proximal (ascending and transverse) colon [34,36]. This heterogeneity in infant microbiota composition has also been observed in other fermentation studies with several infant donors [25,26,27,28,31,32]. To reduce the variability across donors, we included only donors who had not started solid foods and were, at least in part, breast-fed.

In this study, all three infants who donated faecal samples for the inoculum had relatively high Actinobacteria (and/or *Bifidobacterium*) levels. On average, at the phylum level, the microbiota composition of the three inocula in our study were comparable with those of healthy, term, breast-fed 1–4-month-old infants from Ireland and the US [58,59], although a Spanish study has reported bacterial inocula composition from six infants with much higher relative abundances of Proteobacteria [25]. However, in a more detailed analysis, large differences in the microbiota composition of the inocula were observed between the three donors. The genera *Blautia* and *C. aerofaciens* were detected in the inoculum from Donor I but absent from Donors II and III. In addition, there were more diverse *Lactobacillus* species in the inoculum from Donor III versus the other two donors. Further, their abundance of *Bifidobacterium* also differed. Whereas *B. longum/breve* was present in all three donors, Donor I was the only microbiota that contained *B. adolescentis*, and only Donors I and III harboured *B. catenulatum/gallicum*. Similarly, Lawson et al. noted differences in bifidobacterial community and function between three healthy full-term infants (aged 3–5 months) [60].

The use of an in vitro system to study the effects of *B. infantis* Bi-26 and HMOs on the microbiota allows vessels that represent various section of the colon to be sampled, which would otherwise be invasive if taken from a live subject. Although the microbiota composition evolved during simulations, the differences in donor microbiota compositions remained both in control simulations and with HMO and *B. infantis* Bi-26 treatments. The disparities in HMO consumption and fucose levels between simulations could have resulted from differences in the faecal microbiota compositions between donor infants. Samples of the simulated microbiota with Donor II contained no *B. catenulatum/gallicum*. *B adolescentis*, *C. aerofaciens*, and the genera *Lachnospira*, *Blautia*, *Dorea*, and *Sutterella* were present only in simulations with Donor I, whereas *B. animalis* and *V. parvula* were primarily present in the simulation with Donor II. The simulation with Donor III had relative abundance of *L. gasserii/johnsonii* of 9% to 63% between vessels and treatments, and it was the only simulation to contain *L. salivarius* and *L. vaginalis.* Few articles have reported the microbiota composition of individual donors.

The α-diversity was similar in all simulations, and all HMO treatments decreased the α-diversity compared with the control simulations. In all simulations, the addition of 2′-FL, 3-FL, and 2′-FL + 3-FL resulted in increase in the phylum Actinobacteria—specifically, *B. longum/breve* and *B. catenulatum/gallicum*, which was expected, because these are species utilising HMOs [10,22,24,51]. Similarly, 2′-FL and 3-FL increased the total bifidobacterial and *B. infantis* numbers (in the vessels where it was added) versus the control simulations as detected by qPCR. Thus, the addition of HMOs increased those species that utilise HMOs, albeit differentially between individual simulations.

The changes in the composition of infant gut microbiota also influence the production of microbial metabolites, such as SCFAs and BCFAs [61]. Tsukuda et al. followed 12 Japanese infants from birth to 24 months and found variability in the microbiota composition that was mirrored in their metabolite production [62]. The levels of SCFAs and BCFAs in the current study were comparable with our previous work [26], and with the results of a 24 h batch model [53]. A trend for an increase in acetic acid and SCFA sum was found with 2′-FL and in lactic acid with both 2′-FL and 3-FL. However, when examining how the HMO utilization relates to the production of fucose, SCFA, and BCFA, we noted a clear relationship between 2′-FL and 3-FL utilisation by the microbiota and higher SCFA and SCFA + lactate levels. This might indicate that HMO utilisation by the microbiota could translate into greater increased metabolite production. Similarly, 2′-FL upregulated acetate and lactate levels, corresponding to a rise in the relative abundance of *Bifidobacterium* and bifidobacterial numbers. *Bifidobacterium* species are known acetate and lactate producers [5,56,62]. Simulation studies enable samples to be drawn from various sections of the artificial colon, but they often do not consider the absorption of metabolites. The comparison of simulation studies with faecal SCFA levels is also complicated, because in faecal samples, SCFA levels comprise the sum of the production, absorption, and consumption by the surrounding microbiota and transit time [61].

The strengths of this study include its comparison of HMOs, individually and in combination, with and without HMO-utilising *B. infantis* Bi-26 bacterium and infant inocula in parallel in an in vitro colon model. Consequently, we could distinguish the effects of HMOs on individual microbiotas and analyse individual variations between the three donors. Conversely, this setup was a limitation, allowing us to draw conclusions solely from these three individual simulations. Because all three donors had relatively high *Bifidobacterium* levels from the outset of the simulation, the addition of *B. infantis* Bi-26 induced only minor changes. To have better detected the possible benefits of *B. infantis* Bi-26 on infant microbiota composition and metabolites, we would have had to pre-screen donors with lower *Bifidobacterium* levels or added more *B. infantis* Bi-26. Moreover, the microbiota composition in in vitro colon models is based on faecal inoculum, not actual colonic microbiota. To obtain sufficient faecal material for the seven parallel simulations, we also had to combine samples from a single donor within a maximum of 2 weeks. Due to the paucity of in vivo data in infants, discrepancies between other models, and interindividual variation, we did not alter pH, duration of the semi-continuous fermentation, or nutrient composition. In future studies, changes to these parameters, the addition of a mucosal component, and absorption of the nutrients could improve the in vitro modelling of fermentation by infant microbiota.

## 5. Conclusions

In conclusion, this in vitro model is a suitable alternative that can be used to increase our understanding of the effects of 2′-FL and 3-FL on microbial composition and metabolism. In our study, these HMOs increased the relative abundance and numbers of *Bifidobacterium* species that can ferment them. In particular, 2′-FL and 3-FL influence the intestinal microbiota composition in an individual manner. HMO utilisation correlated with greater SCFA and lactate production. At the species level, we noted differences in microbiota composition between donors. This study highlights the importance of better understanding the changes in the composition of individual infant microbiota and the effects of HMOs and probiotics on them.

## Figures and Tables

**Table 1:** Levels of (a) 2′-fucosyllactose (2′-FL, %) and fucose (mg/L) and (b) 3-fucosyllactose (3-FL, %) and fucose (mg/L) in the simulation vessels after 48 h of simulation. The 2′-FL + 3-FL combination was added as half 2′-FL and half 3-FL. Fucose values from the 2′-FL + 3-FL combination vessels are highlighted in light grey. Bi-26 = *Bifidobacterium longum* subspecies *infantis* Bi-26; nd = not detected.

**(a)**		2′-FL Added					2′-FL + 3-FL Added							2′-FL + Bi-26 Added			
		Vessel					Vessel							Vessel			
Donor		1	2		3	4	1	2		3		4		1	2	3	4
I	2′-FL	1.72	0.32		nd	nd	0.79	0.08		nd		nd		1.77	1.59	1.39	0.32
	Fucose	148	2495		nd	nd	371	3495		70		nd		225	167	353	612
II	2′-FL	1.77	nd		nd	nd	0.87	0.85		0.72		0.28		1.75	0.52	0.12	nd
	Fucose	nd	nd		nd	nd	nd	nd		nd		237		nd	164	117	2
III	2′-FL	1.76	1.47		0.35	nd	0.84	0.68		nd		nd		0.6	nd	nd	nd
	Fucose	nd	321		338	nd	nd	149		nd		nd		1261	1760	nd	nd
**(b)**		3-FL added					2′-FL + 3-FL added							3-FL + Bi-26 added			
		Vessel					Vessel							Vessel			
Donor		1		2	3	4	1		2		3		4	1	2	3	4
I	3-FL	0.51		nd	nd	nd	0.82		0.52		nd		nd	1.76	1.71	0.99	0.11
	Fucose	1515		706	nd	nd	371		3495		70		nd	61	33	134	107
II	3-FL	1.6		1.59	1.39	0.98	0.82		0.78		0.59		0.09	0.27	0.22	nd	nd
	Fucose	nd		nd	nd	nd	nd		nd		nd		237	48	69	6	nd
III	3-FL	1.76		1.67	1.54	nd	0.84		0.74		nd		nd	1.8	1.78	0.63	nd
	Fucose	nd		nd	241	nd	nd		149		nd		nd	nd	nd	141	nd

## Data Availability

Data are contained within the article and supplementary material.

## Supplementary Materials

The following supporting information can be downloaded at: https://www.mdpi.com/article/10.3390/microorganisms11061553/s1, Supplementary material including: Supplementary Table S1: Bacterial composition of the inocula; Supplementary Table S3: Total bacterial cell numbers; Supplementary Figure S1: Alpha diversity of the simulation samples according to group; Supplementary Figure S2: Alpha diversity of the simulation samples according to probiotic status; Supplementary Figure S3: Correlation between human milk oligosaccharides and fucose; and an Excel Supplementary Table S2: 16S rRNA amplicon sequencing.

## References

[1] Saturio S., Nogacka A.M., Suárez M., Fernández N., Mantecón L., Mancabelli L., Milani C., Ventura M., de los Reyes-Gavilán C.G., Solís G. (2021). Early-Life Development of the Bifidobacterial Community in the Infant Gut. Int. J. Mol. Sci..

[2] Masi A.C., Stewart C.J. (2022). Untangling human milk oligosaccharides and infant gut microbiome. iScience.

[3] Milani C., Duranti S., Bottacini F., Casey E., Turroni F., Mahony J., Belzer C., Delgado Palacio S., Arboleya Montes S., Mancabelli L. (2017). The First Microbial Colonizers of the Human Gut: Composition, Activities, and Health Implications of the Infant Gut Microbiota. Microbiol. Mol. Biol. Rev..

[4] Chong C.Y.L., Bloomfield F.H., O’Sullivan J.M. (2018). Factors Affecting Gastrointestinal Microbiome Development in Neonates. Nutrients.

[5] Stuivenberg G.A., Burton J.P., Bron P.A., Reid G. (2022). Why Are Bifidobacteria Important for Infants?. Microorganisms.

[6] Saturio S., Nogacka A.M., Alvarado-Jasso G.M., Salazar N., de Los Reyes-Gavilán C.G., Gueimonde M., Arboleya S. (2021). Role of Bifidobacteria on Infant Health. Microorganisms.

[7] Boudry G., Charton E., Le Huerou-Luron I., Ferret-Bernard S., Le Gall S., Even S., Blat S. (2021). The Relationship Between Breast Milk Components and the Infant Gut Microbiota. Front. Nutr..

[8] Engfer M.B., Stahl B., Finke B., Sawatzki G., Daniel H. (2000). Human milk oligosaccharides are resistant to enzymatic hydrolysis in the upper gastrointestinal tract. Am. J. Clin. Nutr..

[9] Li Y., Faden H.S., Zhu L. (2020). The Response of the Gut Microbiota to Dietary Changes in the First Two Years of Life. Front. Pharmacol..

[10] Salli K., Hirvonen J., Siitonen J., Ahonen I., Anglenius H., Maukonen J. (2021). Selective Utilization of the Human Milk Oligosaccharides 2′-Fucosyllactose, 3-Fucosyllactose, and Difucosyllactose by Various Probiotic and Pathogenic Bacteria. J. Agric. Food Chem..

[11] Yu Z.T., Chen C., Newburg D.S. (2013). Utilization of major fucosylated and sialylated human milk oligosaccharides by isolated human gut microbes. Glycobiology.

[12] Samuel T.M., Zhou Q., Giuffrida F., Munblit D., Verhasselt V., Thakkar S.K. (2020). Nutritional and Non-nutritional Composition of Human Milk Is Modulated by Maternal, Infant, and Methodological Factors. Front. Nutr..

[13] Daniels V.C., Monaco M.H., Wang M., Hirvonen J., Jensen H.M., Ouwehand A.C., Mukherjea R., Dilger R.N., Donovan S.M. (2021). Evaluation of 2′-Fucosyllactose and Bifidobacterium longum Subspecies infantis on Growth, Organ Weights, and Intestinal Development of Piglets. Nutrients.

[14] Marriage B.J., Buck R.H., Goehring K.C., Oliver J.S., Williams J.A. (2015). Infants Fed a Lower Calorie Formula with 2′-fucosyllactose (2′FL) Show Growth and 2′FL Uptake Like Breast-Fed Infants. J. Pediatr. Gastroenterol. Nutr..

[15] Storm H.M., Shepard J., Czerkies L.M., Kineman B., Cohen S.S., Reichert H., Carvalho R. (2019). 2′-Fucosyllactose Is Well Tolerated in a 100% Whey, Partially Hydrolyzed Infant Formula With *Bifidobacterium lactis*: A Randomized Controlled Trial. Glob. Pediatr. Health.

[16] Alliet P., Vandenplas Y., Roggero P., Jespers S.N.J., Peeters S., Stalens J.P., Kortman G.A.M., Amico M., Berger B., Sprenger N. (2022). Safety and efficacy of a probiotic-containing infant formula supplemented with 2′-fucosyllactose: A double-blind randomized controlled trial. Nutr. J..

[17] Puccio G., Alliet P., Cajozzo C., Janssens E., Corsello G., Sprenger N., Wernimont S., Egli D., Gosoniu L., Steenhout P. (2017). Effects of Infant Formula With Human Milk Oligosaccharides on Growth and Morbidity: A Randomized Multicenter Trial. J. Pediatr. Gastroenterol. Nutr..

[18] Bosheva M., Tokodi I., Krasnow A., Pedersen H.K., Lukjancenko O., Eklund A.C., Grathwohl D., Sprenger N., Berger B., Cercamondi C.I. (2022). Infant Formula With a Specific Blend of Five Human Milk Oligosaccharides Drives the Gut Microbiota Development and Improves Gut Maturation Markers: A Randomized Controlled Trial. Front. Nutr..

[19] Lasekan J., Choe Y., Dvoretskiy S., Devitt A., Zhang S., Mackey A., Wulf K., Buck R., Steele C., Johnson M. (2022). Growth and Gastrointestinal Tolerance in Healthy Term Infants Fed Milk-Based Infant Formula Supplemented with Five Human Milk Oligosaccharides (HMOs): A Randomized Multicenter Trial. Nutrients.

[20] Parschat K., Melsaether C., Jäpelt K.R., Jennewein S. (2021). Clinical Evaluation of 16-Week Supplementation with 5HMO-Mix in Healthy-Term Human Infants to Determine Tolerability, Safety, and Effect on Growth. Nutrients.

[21] Berger B., Porta N., Foata F., Grathwohl D., Delley M., Moine D., Charpagne A., Siegwald L., Descombes P., Alliet P. (2020). Linking Human Milk Oligosaccharides, Infant Fecal Community Types, and Later Risk To Require Antibiotics. mBio.

[22] Thongaram T., Hoeflinger J.L., Chow J., Miller M.J. (2017). Human milk oligosaccharide consumption by probiotic and human-associated bifidobacteria and lactobacilli. J. Dairy Sci..

[23] Zabel B., Yde C.C., Roos P., Marcussen J., Jensen H.M., Salli K., Hirvonen J., Ouwehand A.C., Morovic W. (2019). Novel Genes and Metabolite Trends in *Bifidobacterium longum* subsp. *infantis* Bi-26 Metabolism of Human Milk Oligosaccharide 2′-fucosyllactose. Sci. Rep..

[24] Zabel B.E., Gerdes S., Evans K.C., Nedveck D., Singles S.K., Volk B., Budinoff C. (2020). Strain-specific strategies of 2′-fucosyllactose, 3-fucosyllactose, and difucosyllactose assimilation by *Bifidobacterium longum* subsp. *infantis* Bi-26 and ATCC 15697. Sci. Rep..

[25] Nogacka A.M., Arboleya S., Nikpoor N., Auger J., Salazar N., Cuesta I., Mantecón L., Solís G., Gueimonde M., Tompkins T.A. (2021). Influence of 2′-Fucosyllactose on the Microbiota Composition and Metabolic Activity of Fecal Cultures from Breastfed and Formula-Fed Infants at Two Months of Age. Microorganisms.

[26] Salli K., Anglenius H., Hirvonen J., Hibberd A.A., Ahonen I., Saarinen M.T., Tiihonen K., Maukonen J., Ouwehand A.C. (2019). The effect of 2′-fucosyllactose on simulated infant gut microbiome and metabolites; a pilot study in comparison to GOS and lactose. Sci. Rep..

[27] Van den Abbeele P., Duysburgh C., Vazquez E., Chow J., Buck R., Marzorati M. (2019). 2′-Fucosyllactose alters the composition and activity of gut microbiota from formula-fed infants receiving complementary feeding in a validated intestinal model. J. Funct. Foods.

[28] Van den Abbeele P., Sprenger N., Ghyselinck J., Marsaux B., Marzorati M., Rochat F. (2021). A Comparison of the In Vitro Effects of 2′Fucosyllactose and Lactose on the Composition and Activity of Gut Microbiota from Infants and Toddlers. Nutrients.

[29] Natividad J.M., Marsaux B., Rodenas C.L.G., Rytz A., Vandevijver G., Marzorati M., Van den Abbeele P., Calatayud M., Rochat F. (2022). Human Milk Oligosaccharides and Lactose Differentially Affect Infant Gut Microbiota and Intestinal Barrier In Vitro. Nutrients.

[30] Kong C., Akkerman R., Klostermann C.E., Beukema M., Oerlemans M.M.P., Schols H.A., de Vos P. (2021). Distinct fermentation of human milk oligosaccharides 3-FL and LNT2 and GOS/inulin by infant gut microbiota and impact on adhesion of Lactobacillus plantarum WCFS1 to gut epithelial cells. Food Funct..

[31] Wiese M., Khakimov B., Nielsen S., Sørensen H., van den Berg F., Nielsen D.S. (2018). CoMiniGut-a small volume in vitro colon model for the screening of gut microbial fermentation processes. PeerJ.

[32] Nogacka A.M., Arboleya S., Nikpoor N., Auger J., Salazar N., Cuesta I., Alvarez-Buylla J.R., Mantecón L., Solís G., Gueimonde M. (2022). In Vitro Probiotic Modulation of the Intestinal Microbiota and 2′Fucosyllactose Consumption in Fecal Cultures from Infants at Two Months of Age. Microorganisms.

[33] Martín-Peláez S., Cano-Ibáñez N., Pinto-Gallardo M., Amezcua-Prieto C. (2022). The Impact of Probiotics, Prebiotics, and Synbiotics during Pregnancy or Lactation on the Intestinal Microbiota of Children Born by Cesarean Section: A Systematic Review. Nutrients.

[34] Mäkeläinen H., Ottman N., Forssten S., Saarinen M., Rautonen N., Ouwehand A.C. (2010). Synbiotic effects of GOS, PDX and Bifidobacterium lactis Bi-07 in vitro. Int. J. Probiotics Prebiotics.

[35] Mäkeläinen H.S., Mäkivuokko H.A., Salminen S.J., Rautonen N.E., Ouwehand A.C. (2007). The effects of polydextrose and xylitol on microbial community and activity in a 4-stage colon simulator. J. Food Sci..

[36] Mäkivuokko H., Nurmi J., Nurminen P., Stowell J., Rautonen N. (2005). In vitro effects on polydextrose by colonic bacteria and caco-2 cell cyclooxygenase gene expression. Nutr. Cancer.

[37] Mäkivuokko H.A., Saarinen M.T., Ouwehand A.C., Rautonen N.E. (2006). Effects of lactose on colon microbial community structure and function in a four-stage semi-continuous culture system. Biosci. Biotechnol. Biochem..

[38] Mäkivuokko H., Kettunen H., Saarinen M., Kamiwaki T., Yokoyama Y., Stowell J., Rautonen N. (2007). The effect of cocoa and polydextrose on bacterial fermentation in gastrointestinal tract simulations. Biosci. Biotechnol. Biochem..

[39] Apajalahti J.H., Kettunen H., Kettunen A., Holben W.E., Nurminen P.H., Rautonen N., Mutanen M. (2002). Culture-independent microbial community analysis reveals that inulin in the diet primarily affects previously unknown bacteria in the mouse cecum. Appl. Environ. Microbiol..

[40] Mäkeläinen H., Saarinen M., Stowell J., Rautonen N., Ouwehand A.C. (2010). Xylo-oligosaccharides and lactitol promote the growth of *Bifidobacterium lactis* and *Lactobacillus* species in pure cultures. Benef. Microbes.

[41] Lehtoranta L., Hibberd A.A., Reimari J., Junnila J., Yeung N., Maukonen J., Crawford G., Ouwehand A.C. (2020). Recovery of Vaginal Microbiota After Standard Treatment for Bacterial Vaginosis Infection: An Observational Study. Microorganisms.

[42] Bolyen E., Rideout J.R., Dillon M.R., Bokulich N.A., Abnet C.C., Al-Ghalith G.A., Alexander H., Alm E.J., Arumugam M., Asnicar F. (2019). Reproducible, interactive, scalable and extensible microbiome data science using QIIME 2. Nat. Biotechnol..

[43] DeSantis T.Z., Hugenholtz P., Larsen N., Rojas M., Brodie E.L., Keller K., Huber T., Dalevi D., Hu P., Andersen G.L. (2006). Greengenes, a chimera-checked 16S rRNA gene database and workbench compatible with ARB. Appl. Environ. Microbiol..

[44] Ouwehand A.C., Tiihonen K., Saarinen M., Putaala H., Rautonen N. (2009). Influence of a combination of *Lactobacillus acidophilus* NCFM and lactitol on healthy elderly: Intestinal and immune parameters. Br. J. Nutr..

[45] Faith D.P. (1992). Conservation evaluation and phylogenetic diversity. Biol. Conserv..

[46] Benjamini Y., Hochberg Y. (1995). Controlling the False Discovery Rate: A Practical and Powerful Approach to Multiple Testing. J. R. Stat. Soc. Series B. Stat. Methodol..

[47] Lozupone C., Knight R. (2005). UniFrac: A new phylogenetic method for comparing microbial communities. Appl. Environ. Microbiol..

[48] R Core Team (2022). R: A Language and Environment for Statistical Computing.

[49] Brunner E., Domhof S., Langer F. (2002). Nonparametric Analysis of Longitudinal Data in Factorial Experiments.

[50] Noguchi K., Gel Y.R., Brunner E., Konietschke F. (2012). nparLD: An R Software Package for the Nonparametric Analysis of Longitudinal Data in Factorial Experiments. J. Stat. Softw..

[51] Sakanaka M., Gotoh A., Yoshida K., Odamaki T., Koguchi H., Xiao J.Z., Kitaoka M., Katayama T. (2019). Varied Pathways of Infant Gut-Associated Bifidobacterium to Assimilate Human Milk Oligosaccharides: Prevalence of the Gene Set and Its Correlation with Bifidobacteria-Rich Microbiota Formation. Nutrients.

[52] Fournier E., Roussel C., Dominicis A., Ley D., Peyron M.A., Collado V., Mercier-Bonin M., Lacroix C., Alric M., Van de Wiele T. (2022). In vitro models of gut digestion across childhood: Current developments, challenges and future trends. Biotechnol. Adv..

[53] Ryan J.J., Monteagudo-Mera A., Contractor N., Gibson G.R. (2021). Impact of 2′-Fucosyllactose on Gut Microbiota Composition in Adults with Chronic Gastrointestinal Conditions: Batch Culture Fermentation Model and Pilot Clinical Trial Findings. Nutrients.

[54] Šuligoj T., Vigsnæs L.K., Abbeele P.V.D., Apostolou A., Karalis K., Savva G.M., McConnell B., Juge N. (2020). Effects of Human Milk Oligosaccharides on the Adult Gut Microbiota and Barrier Function. Nutrients.

[55] Vigsnaes L.K., Ghyselinck J., Van den Abbeele P., McConnell B., Moens F., Marzorati M., Bajic D. (2021). 2′FL and LNnT Exert Antipathogenic Effects against C. difficile ATCC 9689 In Vitro, Coinciding with Increased Levels of Bifidobacteriaceae and/or Secondary Bile Acids. Pathogens.

[56] Li H., Lane J.A., Chen J., Lu Z., Wang H., Dhital S., Fu X., Huang Q., Liu F., Zhang B. (2022). In vitro fermentation of human milk oligosaccharides by individual Bifidobacterium longum-dominant infant fecal inocula. Carbohydr. Polym..

[57] Bode L. (2020). Human Milk Oligosaccharides: Structure and Functions. Nestle Nutr. Inst. Workshop Ser..

[58] Hill C.J., Lynch D.B., Murphy K., Ulaszewska M., Jeffery I.B., O’Shea C.A., Watkins C., Dempsey E., Mattivi F., Tuohy K. (2017). Evolution of gut microbiota composition from birth to 24 weeks in the INFANTMET Cohort. Microbiome.

[59] Wang M., Li M., Wu S., Lebrilla C.B., Chapkin R.S., Ivanov I., Donovan S.M. (2015). Fecal microbiota composition of breast-fed infants is correlated with human milk oligosaccharides consumed. J. Pediatr. Gastroenterol. Nutr..

[60] Lawson M.A.E., O’Neill I.J., Kujawska M., Gowrinadh Javvadi S., Wijeyesekera A., Flegg Z., Chalklen L., Hall L.J. (2020). Breast milk-derived human milk oligosaccharides promote Bifidobacterium interactions within a single ecosystem. ISME J..

[61] Łoniewski I., Skonieczna-Żydecka K., Stachowska L., Fraszczyk-Tousty M., Tousty P., Łoniewska B. (2022). Breastfeeding Affects Concentration of Faecal Short Chain Fatty Acids During the First Year of Life: Results of the Systematic Review and Meta-Analysis. Front. Nutr..

[62] Tsukuda N., Yahagi K., Hara T., Watanabe Y., Matsumoto H., Mori H., Higashi K., Tsuji H., Matsumoto S., Kurokawa K. (2021). Key bacterial taxa and metabolic pathways affecting gut short-chain fatty acid profiles in early life. ISME J..

