# Supplementary material for: The Effect of Human Milk Oligosaccharides and Bifidobacterium longum subspecies infantis Bi-26 on Simulated Infant Gut Microbiome and Metabolites
Source: Microorganisms. 2023 Jun 10;11(6):1553. doi: 10.3390/microorganisms11061553 (PMC10304344; doi:10.3390/microorganisms11061553)
Supplement: Supplementary file 1 [file microorganisms-11-01553-s001.zip › microorganisms-2421382-supplementary.pdf]

The effect of human milk oligosaccharides and *Bifidobacterium longum* subspecies *infantis* Bi-26 on simulated infant gut microbiome and metabolites

Supplementary Table S1: Bacterial composition of the inocula. The inoculum from each donor with and without the *Bifidobacterium longum* subspecies *infantis* Bi-26 (Bi-26) at the (a) phylum and (b) genus/species levels, with the 10 most abundant species shown.

(a)

	**Donor I**		**Donor II**		**Donor III**	
Phylum	**Without Bi-26**	**With Bi-26**	**Without Bi-26**	**With Bi-26**	**Without Bi-26**	**With Bi-26**
Actinobacteria	71.8%	73.5%	30.8%	55.4%	46.7%	46.1%
Bacteroidetes	1.3%	0.9%	50.5%	28.2%	23.8%	27.6%
Firmicutes	22.0%	22.1%	11.5%	10.2%	18.0%	14.8%
Proteobacteria	2.3%	1.4%	0%	0%	5.6%	5.8%

(b)

	**Donor I**		**Donor II**		**Donor III**	
Species	**Without Bi-26**	**With Bi-26**	**Without Bi-26**	**With Bi-26**	**Without Bi-26**	**With Bi-26**
Bacteroides unknown	0.1%	0.1%	46.9%	26.8%	17.8%	20.1%
Bifidobacterium adolescentis	27.1%	26.2%	0%	0%	0%	0%
Bifidobacterium catenulatum/gallicum	9.2%	9.7%	0%	0%	10.2%	11.0%
Bifidobacterium longum/breve	7.0%	9.5%	30.7%	55.3%	36.5%	35.1%
Blautia unknown	10.6%	11.8%	0%	0%	0%	0%
Collinsella aerofaciens	24.4%	26.2%	0%	0%	0%	0%
Lactobacillus gasseri/johnsonii	0%	0%	0%	0%	9.5%	7.5%
Lactobacilllus zeae	10.1%	11.8%	8.7%	8.9%	4.1%	3.2%
Parabacteroides unknown	0.1%	0.1%	0%	0%	4.6%	5.7%
Veillonella dispar	0.3%	0.0%	1.1%	0.5%	1.8%	1.5%

Supplementary Table S2: 16S rRNA amplicon sequencing: Please upload a separate Excel file

Supplementary Table S3: Total bacterial cell numbers. The total bacterial cell numbers (per ml) after the simulations measured from different vessels analysed by flow cytometry. The table shows the average and standard deviation (std) of the total bacterial cell numbers/ml from three simulations with and without the *Bifidobacterium longum* subspecies *infantis* Bi-26 (Bi-26) and with 2´-fucosyllactose (2´-FL) and 3-fucosyllactose (3-FL), their combination or control (No FL).

Probiotic	Treatment	Vessel	Average cell number/ml	Std
Without Bi-26	Control	1	9.29×10^8	2.09×10^8
		2	1.25×10^9	2.86×10^8
		3	2.75×10^9	4.57×10^8
		4	3.85×10^9	1.04×10^9
	2´-FL	1	6.83×10^9	3.51×10^8
		2	2.37×10^9	1.8×10^9
		3	5.24×10^9	2.17×10^9
		4	5.99×10^9	6.59×10^8
	3-FL	1	1.83×10^9	1.99×10^9
		2	2.44×10^9	2.26×10^9
		3	3.56×10^9	1.77×10^9
		4	5.55×10^9	2.39×10^9
	2´-FL + 3-FL	1	8.85×10^8	3.14×10^8
		2	1.33×10^9	5.62×10^8
		3	3.95×10^9	1.13×10^9
		4	6.13×10^9	8.36×10^7
With Bi-26	Control	1	8.47×10^8	1.04×10^8
		2	1.65×10^9	9.73×10^8
		3	3.18×10^9	1.37×10^9
		4	5.37×10^9	2.16×10^9
	2´-FL	1	1.02×10^9	4.44×10^8
		2	2.78×10^9	2.36×10^9
		3	3.53×10^9	1.64×10^9
		4	5.38×10^9	2.42×10^8
	3-FL	1	3.0×10^9	3.64×10^9
		2	2.5×10^9	2.37×10^9
		3	4.3×10^9	1.68×10^9
		4	6.48×10^9	1.67×10^9

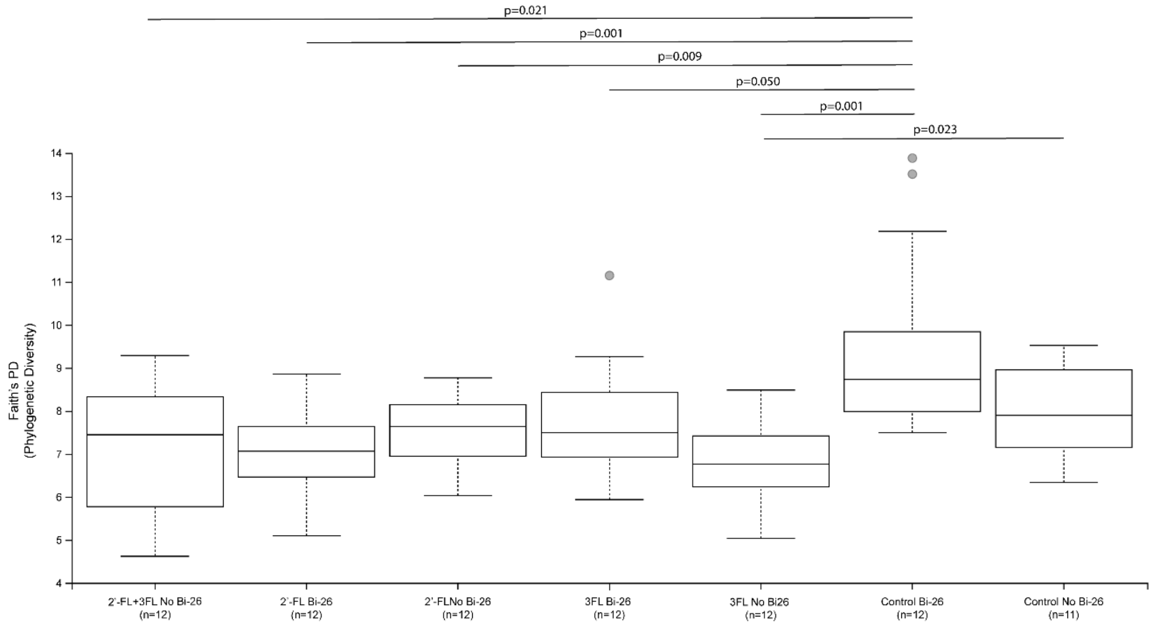

Supplementary Figure S1. Alpha diversity of the simulation samples according to group. Alpha diversity (Faith's phylogenetic diversity metric) between all study groups. Kruskal-Wallis global test p-value= 0.008 and the pairwise post-hoc between-group significance is noted where p<0.05, Wilcoxon rank-sum test.

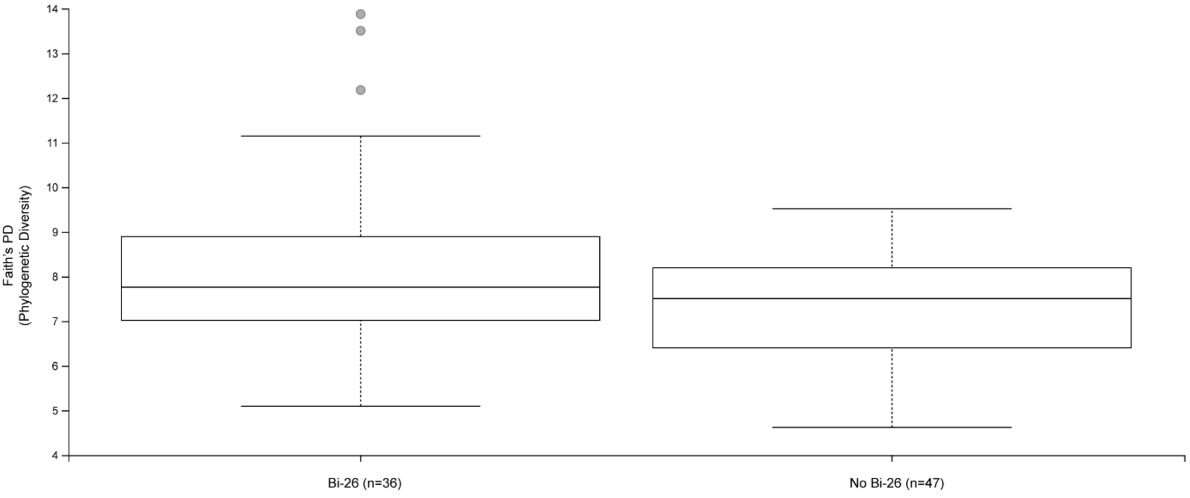

Supplementary Figure S2. Alpha diversity of the simulation samples according to probiotic status; Alpha diversity (Faith's phylogenetic diversity metric) between groups with or without *Bifidobacterium longum* subsp. *infantis* Bi-26 (Bi-26).

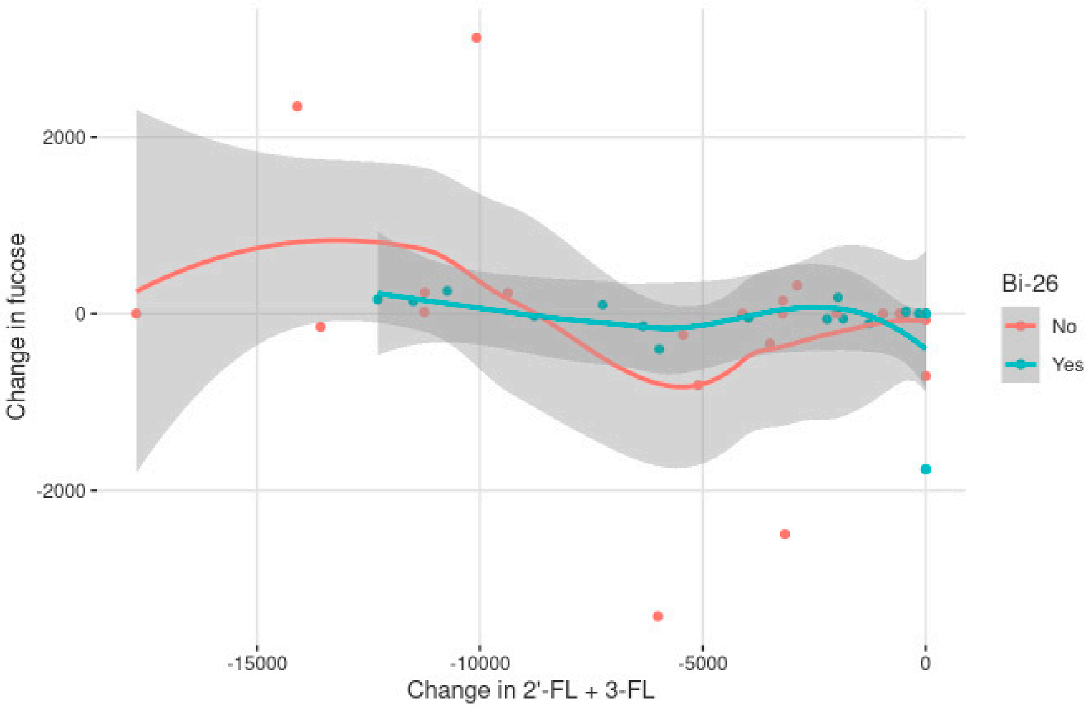

Supplementary Figure S3. Correlation between human milk oligosaccharides and fucose. The correlation between changes in 2´-fucosyllactose (2´-FL) and 3-fucosyllactose (3-FL) and fucose.
